# TSPO PIGA Ligands Promote Neurosteroidogenesis and Human Astrocyte Well-Being

**DOI:** 10.3390/ijms17071028

**Published:** 2016-06-29

**Authors:** Eleonora Da Pozzo, Chiara Giacomelli, Barbara Costa, Chiara Cavallini, Sabrina Taliani, Elisabetta Barresi, Federico Da Settimo, Claudia Martini

**Affiliations:** Department of Pharmacy, University of Pisa, Via Bonanno Pisano 6, 56126 Pisa, Italy; eleonora.dapozzo@unipi.it (E.D.P.); chiara.giacomelli@for.unipi.it (C.G.); barbara.costa@unipi.it (B.C.); chiara.cavallini@farm.unipi.it (C.C.); sabrina.taliani@unipi.it (S.T.); elisabetta.barresi@for.unipi.it (E.B.); federico.dasettimo@unipi.it (F.D.S.)

**Keywords:** translocator protein, neurosteroidogenesis, PIGA ligands, cellular proliferation, oxidative metabolism, astrocytes

## Abstract

The steroidogenic 18 kDa translocator protein (TSPO) is an emerging, attractive therapeutic tool for several pathological conditions of the nervous system. Here, 13 high affinity TSPO ligands belonging to our previously described *N*,*N*-dialkyl-2-phenylindol-3-ylglyoxylamide (PIGA) class were evaluated for their potential ability to affect the cellular Oxidative Metabolism Activity/Proliferation index, which is used as a measure of astrocyte well-being. The most active PIGA ligands were also assessed for steroidogenic activity in terms of pregnenolone production, and the values were related to the metabolic index in rat and human models. The results showed a positive correlation between the increase in the Oxidative Metabolism Activity/Proliferation index and the pharmacologically induced stimulation of steroidogenesis. The specific involvement of steroid molecules in mediating the metabolic effects of the PIGA ligands was demonstrated using aminoglutethimide, a specific inhibitor of the first step of steroid biosynthesis. The most promising steroidogenic PIGA ligands were the 2-naphthyl derivatives that showed a long residence time to the target, in agreement with our previous data. In conclusion, TSPO ligand-induced neurosteroidogenesis was involved in astrocyte well-being.

## 1. Introduction

Neuroactive steroids, which are mainly synthesized by glial cells, exert peculiar actions to influence the development and function of the nervous system through both genomic and non-genomic mechanisms [1,2]. The classic genomic action involves steroid binding to intracellular receptors and the regulation of protein translation [3]. Neuroactive steroids can also show rapid effects, occurring within seconds to minutes, via the activation of membrane neurotransmitter receptors. It has been shown that neuroactive steroids determine the allosteric modulations on ligand-gated channels, including type-A γ-aminobutyric acid (GABA), *N*-methyl-d-aspartate (NMDA), and nicotinic receptors [1,4,5,6,7,8,9,10,11]. These different interactions lead to the multiple actions of neuroactive steroids, affecting both glia and neurons in a concerted manner [12]. For instance, oestrogens act as transcriptional regulators to modulate the synthesis of various proteins and growth factors in astrocytes [13,14,15,16,17,18,19,20,21,22,23,24,25]. Interestingly, oestrogens increase glutamate transporter expression in astrocytes via the nuclear factor κ-light-chain-enhancer of activated B cells (NF-κB) and the cAMP response element binding protein (CREB) pathways [26]. Among the actions exerted by steroids, the increase in the expression of the mitochondria-encoded subunits of the respiratory chain influences the mitochondrial respiratory function, and this activity may be of particular interest for enhancing the functional efficiency of astrocytes [27,28].

Notably, bidirectional glia-neuron communication was suggested by several scientific reports showing that glial cells and neurons can respond to the same signals and that they can mutually modulate the cellular response (for a review see [12]). For this reason, glial cell well-being is of particular importance for the efficiency of the whole brain.

Translocator protein 18 kDa (TSPO), which is primarily located in the outer mitochondrial membrane, is highly expressed in steroid-synthesizing tissues, including glial cells. Although few reports questioned the role of TSPO in steroidogenesis [29,30,31], most studies, including the most recent ones, propose that TSPO is an important protein for steroid synthesis [32,33,34]. TSPO binds cholesterol with high affinity and, in a combined action with the steroidogenic acute regulatory protein (StAR), allows cholesterol to translocate into mitochondria, which represents the rate-limiting step of steroidogenesis [35,36,37]. The steroid biosynthetic pathway is triggered by the cleavage of the cholesterol aliphatic side chain, which is catalyzed by the cytochrome P450 side chain cleavage (P450scc) enzyme, producing pregnenolone. Then, pregnenolone is converted to other neurosteroids by enzymes located in the endoplasmic reticulum, such as hydroxysteroid dehydrogenases [38,39].

A number of TSPO-targeted molecules have been reported as neuroprotective, anti-inflammatory, and regenerating agents in different in vitro and in vivo models, suggesting their possible development as effective therapeutic tools (for a review see [40]). For instance, in gliosis, TSPO ligands were able to decrease reactive gliosis and prevent neuronal loss [41,42]. The stimulation of neurosteroidogenesis has been hypothesized as the basis for the positive actions of the TSPO ligand [43], and, for these reasons, TSPO ligands are currently under investigation as therapeutic tools to preserve a functional brain environment and the glia-neuron bidirectional interactions [44]. Very recently, we have found that the *N*,*N*-dialkyl-2-phenylindol-3-ylglyoxylamide class (PIGAs) of TSPO ligands reduces oxidative stress and the activity of pro-inflammatory enzymes in rat glial cells through the de novo neurosteroid synthesis [45].

Based on the previously described pro-survival activity of TSPO ligands in neurons and glia, in the present work, the effects of the TSPO ligands on astrocyte well-being were assessed by focusing on the involvement of steroidogenesis. Therefore, the residence time of some investigated ligands were also assessed because we have recently shown that the time over which a ligand interacts with TSPO directly affects its steroidogenic efficacy [46]. The human glioblastoma–astrocytoma cell line U87MG and normal human astrocytes were used as cellular models. U87MG cells express the astrocyte cell marker glial fibrillary acidic protein (GFAP) and are widely used as an in vitro astrocyte model [47,48,49,50]. Recent data have shown comparable responses of U87MG cells and primary human astrocytes after inflammatory insult, highlighting the potential use of U87MG cells in drug discovery stages, as it is not feasible to screen compounds in primary human cells [50]. However, data from healthy human astrocytes were crucial for validating TSPO activity under normal conditions. Thus, 13 high affinity, selective TSPO ligands belonging to our previously described PIGA class [51,52] were selected and evaluated for their ability to increase the Oxidative Metabolism Activity/Proliferation index in human astrocyte models. The most promising compounds were then assessed for their steroidogenic activity and residence time. Finally, the relation between oxidative metabolism, proliferation activity, and the induction of neurosteroidogenesis was investigated.

## 2. Results

### 2.1. N,N-Dialkyl-2-phenylindol-3-ylglyoxylamide (PIGA) Ligands Increase the Oxidative Metabolism Activity/Proliferation Index in a Human Astrocyte Model

TSPO expression has previously been established in U87MG cells [53]. To assess the potential effects of the PIGA ligands on the activation of oxidative metabolism, U87MG cells were cultured under serum-reduced growth conditions; serum starvation is a well-known method to arrest the cells in a basal metabolic state (G0/G1 phase) [54,55]. The metabolic activity of the astrocyte models was estimated using the (3-(4,5-dimethylthiazol-2-yl)-5-(3-carboxymethoxyphenol)-2-(4-sulfophenyl)-2*H*-tetrazolium, inner salt) (MTS) assay [56]. This tetrazolium dye can be reduced by the metabolic reducing agents NADH and NADPH to a water-soluble formazan salt; the amount of produced formazan has been considered a marker of the Oxidative Metabolic Activity index [57]. The redox reactions can occur in both the mitochondria and cytosol; in particular, it has been shown that tetrazolium reduction mainly reflects cytosolic redox activity in astroglia and is dependent on glyceraldehyde-3-phosphate dehydrogenase activity [56]. Furthermore, as the reduction of the tetrazolium compound can only be achieved in viable cells, the tetrazolium assay has also been widely used for the quantitative assessment of cellular proliferation for over three decades [56,57].

**Figure 1:**
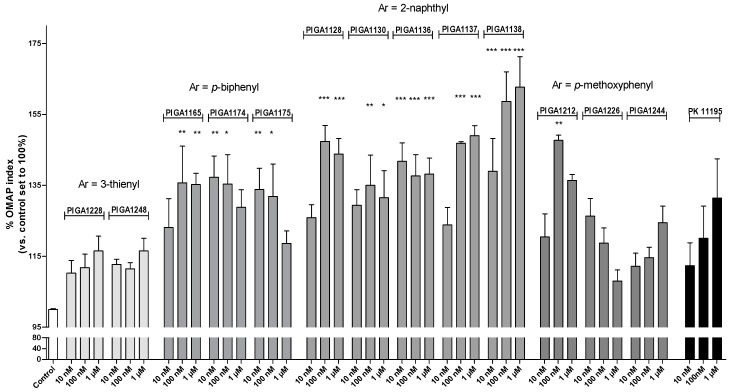
Effects of the PIGA ligands on the Oxidative Metabolism Activity/Proliferation index in U87MG human glioma cells. U87MG cells were treated with different concentrations of the compounds (10 nM–1 µM) in serum-reduced media (1% fetal bovine serum (FBS)), and the [3-(4,5-dimethylthiazol-2-yl)-5-(3-carboxymethoxyphenol)-2-(4-sulfophenyl)-2*H*-tetrazolium, inner salt] (MTS) assay was performed after 48 h of treatment. The data are expressed as percentages of the proliferative/oxidative metabolism activity index compared to the control (0.1% dimethyl sulfoxide (DMSO), a concentration that not interfered with the assay), which was set to 100%, and represent the means ± standard error of the mean (SEM) of three different experiments performed in duplicate. The statistical analysis was performed using one-way analysis of variance (ANOVA) and Bonferroni’s post-test; * *p* < 0.05, ** *p* < 0.01, *** *p* < 0.001 vs. the control.

The effects of PIGAs and the TSPO reference standard ligand PK11195 (ranging from nanomolar to micromolar concentrations) on the Oxidative Metabolism Activity/Proliferation (OMAP) index in U87MG cells were evaluated after 48 h of incubation. The derivatives PIGA1128, PIGA1130, PIGA1136, PIGA1137, PIGA1138, PIGA1165, PIGA1174, PIGA1175, and PIGA1212 significantly increased the OMAP index, with the maximal mean value (163%) observed for 1 µM PIGA1138 (*p* < 0.001 vs. the control) (Figure 1).

The most promising derivatives were those featuring a 2-naphthyl substituent as an aryl group (PIGA1128, PIGA1136, PIGA1137, and PIGA1338; see Table 1 for the chemical structures). In particular, PIGA1136 and PIGA1138 yielded statistically significant results at all tested concentrations. PIGA1174 and PIGA1175 were significantly effective at 10 and 100 nM, the two lowest concentrations tested. Conversely, PIGA1128, PIGA1130, PIGA1137, and PIGA1165 were active at the higher doses of 100 nM and 1 µM. Statistically significant results were not observed for PIGA1226, PIGA1228, PIGA1244, PIGA1248, and the reference compound PK11195. These results suggested that the TSPO ligands positively affected the well-being of an astrocytic cell line when the cells were maintained in a constrained low metabolic state.

### 2.2. PIGA Ligands Effectively Stimulate Steroidogenesis in Vitro

To assess the ability of PIGA ligands to stimulate steroidogenesis in vitro, the synthesis of the first steroid metabolite pregnenolone was evaluated in the presence of inhibitors of pregnenolone metabolism. As a first step, the assessment was performed in a rat C6 cell line, the glial cell model that is conventionally used to measure mitochondrial receptor-regulated steroidogenesis [58]. The amount of pregnenolone released from the C6 cells was measured after a 2 h incubation with a fixed concentration of the most promising PIGAs in terms of metabolic activation (PIGA1128, PIGA1130, PIGA1136, PIGA1137, and PIGA1138).

**Figure 2:**
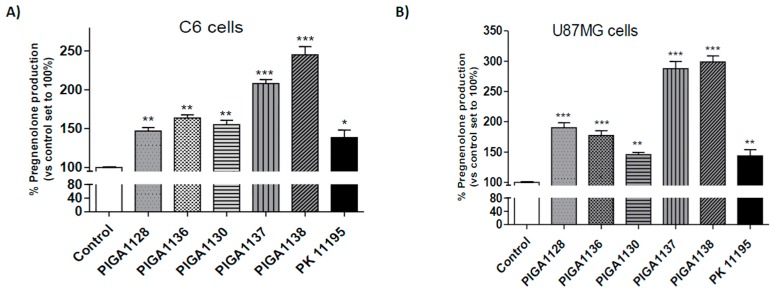
Effects of the PIGA ligands on pregnenolone production. C6 glioma cells (**A**) and U87MG cells (**B**) were incubated with different PIGA ligands (40 µM) for 2 h at 37 °C. The pregnenolone amounts were quantified by a competitive enzyme-linked immunosorbent assay. The data are expressed as percentage of pregnenolone production compared to the control, which was set to 100%, and represent the means ± SEM of three different determinations performed in duplicate. The statistical analysis was performed using one-way ANOVA and Bonferroni’s post-test; * *p* < 0.05, ** *p* < 0.01, *** *p* < 0.001 vs. the control.

The obtained results showed that all PIGA derivatives significantly increased pregnenolone synthesis in the C6 cells compared to the control (cells treated with DMSO and set to 100%) (Figure 2A and Table 2). The highest pregnenolone level was observed after the C6 cells were treated with PIGA1137 and PIGA1138 (increase in pregnenolone synthesis of 208% and 215%, respectively, *p* < 0.001) (Figure 2A and Table 2).

To explore whether the promising steroidogenic effects of the PIGA ligands were maintained in a human astrocytic model, the pregnenolone assessment was performed in U87MG cells. As shown in Figure 2B and Table 2, the PIGA ligands also significantly induced steroidogenesis in the U87MG cells, and the results were comparable to those obtained in the C6 cells. The best performing derivatives were PIGA1137 and PIGA1138, showing an increase in pregnenolone synthesis of 288% and 299%, respectively (*p* < 0.001; Figure 2B and Table 2). Notably, these two PIGA ligands also presented the best metabolic activation profile in the U87MG cells. The standard PK11195 similarly increased pregnenolone production in the C6 and U87MG cells (139% and 144%, respectively, *p* < 0.01) (Figure 2).

### 2.3. The Best Performing PIGA Ligands in Terms of Steroidogenesis Stimulation Are Characterized by a Long Residence Time

Our very recent results have shown that the time over which a ligand interacts with TSPO directly affects its steroidogenic efficacy [46]. The time of the ligand-target interaction is a kinetic parameter known as Residence Time (RT), and it is calculated by the reciprocal of the dissociation rate constant (*k*_off_). For unlabelled TSPO ligands, *k*_off_ is experimentally derived by the competition kinetic association assay [46]. The RT values of the most promising compounds were evaluated to relate the TSPO kinetic binding parameters and steroidogenic activity of the PIGA ligands. Consistent with our previous results, the 2-naphthyl derivative PIGA1138, which is characterized by a long RT (141 min) [46], was the best performing ligand in terms of its ability to stimulate steroidogenesis in both the C6 and U87MG cells. In contrast, the standard PK11195, which is characterized by a short RT (33 min) [46], showed a reduced ability to stimulate steroidogenesis compared to PIGA1138. The RT of PIGA1128 has been already determined (55 min) [46]; as expected, it showed an intermediate ability to stimulate steroidogenesis. The RTs of additional 2-naphthyl derivatives with promising steroidogenic ability in human U87MG cells (PIGA1136 and PIGA1137) were here examined. Kinetic experiments showed that the association rate constant (*k*_on_), *k*_off_ and RT value for PIGA1136 were 2.52 × 10^7^ M^−1^·min^−1^, 0.0178 min^−1^ and 56 min, respectively. For PIGA1137, the *k*_on_, *k*_off_ and RT values were 3.56 × 10^7^ M^−1^·min^−1^, 0.0185 min^−1^ and 54 min, respectively. For PIGA1136 and PIGA1137, the kinetically derived *K*_d_ values (*k*_off_/*k*_on_) were 0.71 and 0.52 nM, respectively. These values were in good agreement with the previously reported *K*_i_ values [52] obtained from competition binding experiments at equilibrium (the *K*_i_ values for PIGA1136 and PIGA1137 were 0.53 and 0.56 nM, respectively).

### 2.4. The Oxidative Metabolism Activity/Proliferation Index of PIGA1138 Is Related to Steroid Production

**Figure 3:**
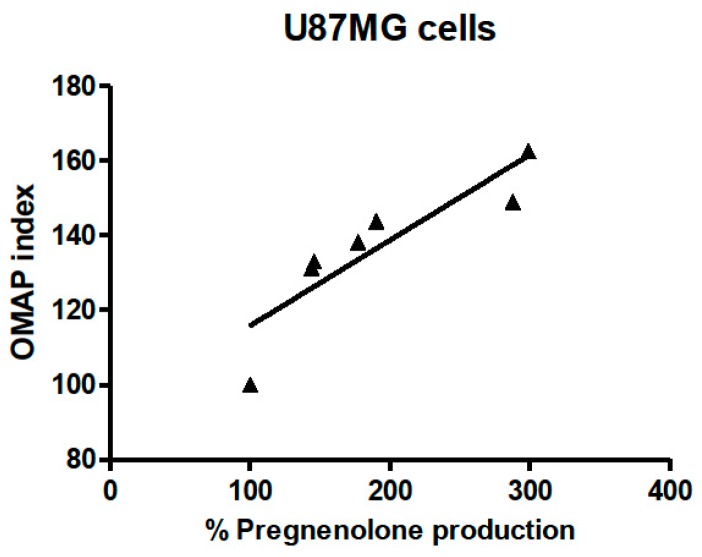
Spearman’s correlation analyses of the Oxidative Metabolism Activity/Proliferation (OMAP) index and pregnenolone production in U87MG cells. The percentages of pregnenolone production obtained in the steroidogenesis experiments (U87MG cells were exposed to 40 µM PIGA ligand in saline medium for 2 h) were correlated to the OMAP indexes obtained in the metabolic experiments (U87MG cells were exposed to 1 µM PIGA ligand in serum-reduced medium for 48 h). The statistical analyses were performed using the Spearman *r* correlation, reporting a *p* < 0.001.

The OMAP index and the percentage of pregnenolone production could not be directly compared, as they were obtained in different experimental settings. However, the correlation analyses indicated a strong relationship between these two parameters. Indeed, Spearman’s correlation analysis of the OMAP index and pregnenolone production in U87MG cells treated with micromolar concentrations of the compounds revealed a highly significant *p* value (*p* = 0.0004, Figure 3).

**Figure 4:**
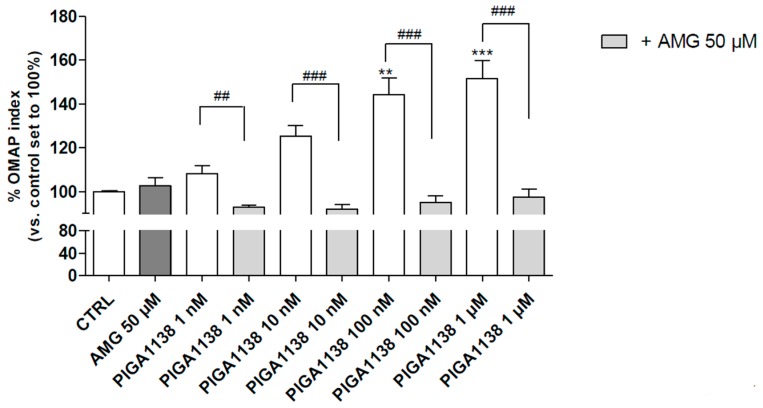
Influence of pregnenolone production on the U87MG OMAP index. U87MG cells were treated with different concentrations of PIGA ligands (1 nM–10 µM) in the absence or presence of AMG (50 µM) in serum-reduced media (1% FBS), and the viable cells were counted after 48 h of treatment using the MTS assay. The data are expressed as percentages of the OMAP index compared to the control, which was set to 100%, and represent the means ± SEM of three different experiments performed in duplicate. The statistical analysis was performed using one-way ANOVA and Bonferroni’s post-test; ** *p* < 0.01, *** *p* < 0.001 vs. the control; ## *p* < 0.01, ### *p* < 0.001 vs. the respective treatment without dl-aminoglutethimide (AMG).

To deeply investigate the correlation between the ability of the most promising PIGA ligands to promote astrocyte survival and their neurosteroidogenic activity, the ligand-mediated metabolic activation of U87MG cells was evaluated in the presence and absence of dl-aminoglutethimide (AMG), an inhibitor of cytochrome P450 side chain cleavage (P450scc), the enzyme that catalyzes the first step of steroidogenesis. The assay was performed for PIGA1138, which was selected as the most representative compound based on its ability to effectively increase both the OMAP index and pregnenolone production. The cells were maintained in a serum-reduced growth condition; in this basal metabolic state, AMG alone did not affect the OMAP index (Figure 4).

The results showed that PIGA1138 increased the OMAP index of the U87MG cells in a concentration-dependent manner and the effects were completely counteracted by the co-treatment with AMG (Figure 4), clearly supporting the hypothesis that the PIGA ligand-induced increase in the OMAP index was mainly related to steroid production.

### 2.5. PIGA1138 Promoted the Activation of Oxidative Metabolism in Normal Human Astrocytes

Although the U87MG cell line is widely used as an astrocyte model in vitro, we verified the consistency of the obtained data by evaluating the effects of PIGA1138 and PK11195 on healthy normal human astrocytes. As first step, the TSPO expression levels were quantified using [^3^H]PK11195 as a probe. In the whole membranes derived from normal human astrocytes, the [^3^H]PK11195 binding reached saturation, showing a maximal binding capacity of 9.548 fmol/mg. In terms of the equilibrium dissociation constant, the experimentally derived [^3^H]PK11195 binding affinity was 2.8 nM.

**Figure 5:**
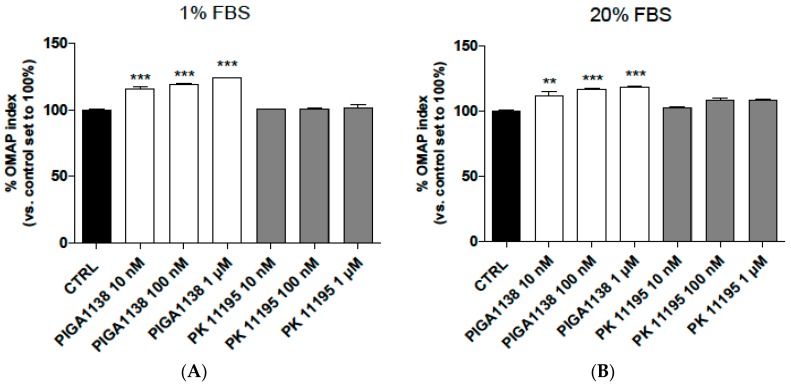
In vitro response of the human astrocytes to the PIGA1138 and PK11195 treatments. (**A**) Human astrocytes were treated with different concentrations of PIGA1138 and PK11195 (10 nM–1 µM) in serum-reduced media (1% FBS), and the MTS assay was performed after 48 h of treatment; (**B**) Human astrocytes were treated with different concentrations of PIGA1138 and PK11195 (10 nM–1·µM) in complete medium and the MTS assay was performed after 48 h of treatment. The data are expressed as percentages of metabolic activity compared to the control, which was set to 100%, and represent the means ± SEM of three different experiments performed in duplicate. ** *p* < 0.01, *** *p* < 0.001 vs. the control.

Then, the effects of PIGA1138 and PK11195 on healthy normal human astrocytes maintained under serum-reduced growth conditions were evaluated (Figure 5A). PK11195 did not increase the astrocytes’ OMAP index. In contrast, PIGA1138 significantly promoted the astrocytes’ well-being (*p* < 0.001) (Figure 5A), in accordance with the data obtained in the U87MG cells (Figure 1).

Finally, to verify if the serum-reduced conditions used in the experiments could affect the obtained results, parallel cell cultures maintained under normal growth conditions (20% serum medium) were treated with ligand and assessed. As shown in Figure 5B, PIGA1138 also increased the astrocytes’ OMAP index under the normal growth culture conditions, whereas PK11195 did not affect the astrocytes’ well-being. The results obtained for PIGA1138 are in accordance with those obtained in astrocytes grown under serum-reduced conditions, suggesting that the increase in steroid production exerted a positive effect when the cells were maintained in a housekeeping metabolic state and promoted the general well-being of the astrocytes under normal growth conditions.

## 3. Discussion

In this study, TSPO PIGA ligand treatments showed a positive relation between steroidogenesis induction and the Oxidative Metabolism Activity/Proliferation Index in rat and human cell models. Notably, the data obtained in these astrocytic cell models were consistent with those acquired in healthy human astrocytes. Firstly, the ability of a number of PIGA ligands to stimulate the OMAP index was shown. Then, the ability of the ligands to induce steroidogenesis was evaluated and related with their TSPO residence time. It is likely that the metabolic stimulation was mediated by the modulation of steroid levels, as the positive effects of the compounds were completely counteracted by the treatment with the neurosteroid synthesis inhibitor AMG.

As previously reported in the nervous system, neuroactive steroids exert several direct regulatory activities on neurons and glial cells [59,60,61,62,63,64,65,66]; the role of TSPO in the release of neurosteroids led us to investigate the relation among the TSPO compound binding ability, OMAP index, and neurosteroid release in rat and human astrocytes. We selected a number of previously reported PIGA ligands with high affinities towards TSPO. The PIGA-induced increase in the OMAP index showed a good agreement with previous data reporting that three different TSPO ligands (triakontatetraneuropeptide, octadecaneuropeptide and Ro5-4864), at low concentrations, induce a dose-dependent increase in DNA synthesis in rat primary astrocytes by activating TSPO [67,68]. These results provide the evidence of a role for TSPO ligands in the control of glial cell proliferation. Indeed, although some forerunner studies have shown that low ligand concentrations inhibit astrocyte proliferation [69,70], most studies have shown a proliferative role for TSPO ligands. For instance, TSPO-related proliferation has been evaluated in C6 glioma cells in serum-free medium, as well as in a standard fibroblast cell line, showing that nanomolar concentrations of PK11195 and Ro5-4864 increased the growth rate and [^3^H]thymidine incorporation [71]. A significant increase in [^3^H]thymidine incorporation in human glioma cells following treatment with 10 nM PK11195 in serum-free media has been confirmed; the same study also showed an increase in mitochondrial mass and lipid fluidity [72]. It has been suggested that the changes in mitochondrial lipid metabolism might lead to mitochondrial biogenesis to support the increased metabolic requirements for cell division [72,73,74].

Concerning the well-known discrepancy between the functional efficacy and affinity of TSPO ligands [75], the PIGA compounds used here presented different functional effects on astrocytes in terms of the OMAP index, despite their similar TSPO affinities. Interestingly, the PIGA compounds that were able to interact with TSPO for a longer time (high RT) also presented higher pregnenolone synthesis induction and promoted better astrocyte well-being, in accordance with our previous data suggesting that the residence time is a predictive parameter for estimating steroidogenic activity [46]. Consistent with the present findings, it has been shown that primary astrocytes and C6 cells that were treated with nanomolar concentrations of PK11195 and Ro5-4864 exhibited an increase in the progesterone content in the medium that was 2–3-fold higher than the basal levels [76]. Similarly, the TSPO ligand AC-5216 increased the allopregnanolone level in meningioma cells [77], supporting the theory that TSPO ligands have a role in the regulation of steroid production. To our knowledge, this is the first study investigating steroidogenesis in a rat cellular model and in human glial cells in parallel, showing a good agreement between the data obtained in these two different cellular models.

Steroidogenesis stimulation is a widely proposed mechanism for the neuroprotective actions of TSPO ligands (for a review see [40]). In this line, we have recently demonstrated the involvement of steroidogenesis in the pro-survival properties of the PIGA ligands against cytotoxic insults, such as lipid peroxidation (induced by cellular glutathione depletion) and inflammatory responses (induced by LPS/IFN-γ cell exposure) [45]. Moreover, the PIGA-elicited modulation of the StAR protein levels has been recently demonstrated; in particular, we have shown that the stimulation of astroglial-derived cells with PIGAs leads to an increase of the 30 kDa intra-mitochondrial StAR, an indirect evidence of an increased cholesterol transfer into mitochondria [45].

It is well known that the effects of steroids are mediated by changes in cellular metabolism [78]. Here, we clearly showed that the positive effects of the PIGA ligands on the astrocytes were mediated by steroids, as they were completely prevented by the pre-treatment with the inhibitor of steroid synthesis, AMG. These data support the theory that the autocrine effects are due to neurosteroid release by the astrocytes themselves.

In conclusion, the importance of the induction of neurosteroidogenesis on astrocyte well-being was investigated in a human astrocyte model in vitro. Astrocytes play a pivotal role in the complex central nervous system network. The loss or the gain of astrocyte functions could be the basis of several pathological conditions [79,80,81,82,83,84,85,86]. In this respect, the positive effects of TSPO-stimulated neurosteroid release on astrocytes well-being were demonstrated. The development of molecules able to stimulate steroid release could represent a therapeutic strategy for central nervous system diseases characterized by astrocyte loss. Furthermore, these ligands may be exploited as pharmacological tools to deeply investigate the autocrine/paracrine roles of neurosteroids in the control of astrocyte metabolism.

## 4. Experimental Section

### 4.1. Materials

[^3^H] PK11195 (Specific Activity, 85.7 μCi/nmol) was obtained from Perkin-Elmer Life Sciences (Perkin Elmer Italia, Monza, Italy). PK11195 and the protease inhibitors were purchased from Sigma-Aldrich (Sigma-Aldrich S.r.l., Milan, Italy). Dulbecco’s Modified Eagle’s Medium, fetal bovine serum, l-glutamine, penicillin, and streptomycin were purchased from Lonza (Milan, Italy). The enzyme-linked immunosorbent assay (ELISA) used to measure the pregnenolone levels was obtained from IBL (Hamburg, Germany). SU10603 and trilostane were gifts from Novartis Farma (Varese, Italy) and Susanne Zister (University of Dublin, Dublin, Ireland), respectively. All other chemical reagents were obtained from commercial sources.

### 4.2. Drugs

The compounds PIGA1228, PIGA1248, PIGA1175, PIGA1165, PIGA1174, PIGA1128, PIGA1136, PIGA1130, PIGA1137, PIGA1138, PIGA1226, PIGA1244, and PIGA1212 were synthesized according to the experimental procedure that we previously described [51,52]. Briefly, the appropriate 2-arylindoles, which were commercially available or easily obtained with a one-step Fischer indole synthesis, were reacted with oxalyl chloride at room temperature in anhydrous diethyl ether to produce the corresponding 2-arylindolylglyoxylyl chlorides. These compounds were then treated with the appropriate dialkylamine in dry toluene solution in the presence of triethylamine at room temperature to yield the target PIGA ligands [51,52].

### 4.3. Cell Culture

U87MG cells were purchased from the National Institute for Cancer Research of Genoa (Genoa Italy) and cultured in RPMI supplemented with 10% fetal bovine serum (FBS), 2 mM l-glutamine, 1% non-essential amino acids, penicillin (100 U/mL) and streptomycin (100 mg/mL) at 37 °C in 5% CO_2_. C6 rat glioma cells were cultured in Dulbecco’s Modified Eagle’s Medium (DMEM) supplemented with 10% FBS, 2 mM l-glutamine, penicillin (100 U/mL) and streptomycin (100 mg/mL) at 37 °C in 5% CO_2_. Human astrocytes were obtained from GIBCO (Life Technologies, Milan, Italy). Human astrocytes were cultured in DMEM supplemented with 10% FBS, 1% non-essential amino acids, 1% N-2 Supplement, penicillin (100 U/mL) and streptomycin (100 mg/mL) at 37 °C in 5% CO_2_.

### 4.4. The Oxidative Metabolism Activity/Proliferation Index in the Astrocyte Cell Models

Rat C6 cells, human U87MG cells or human primary astrocytes were seeded in 96-well plates (10,000 cells/well) and maintained in their specific, complete culture media for 24 h. Then, the culture media were refreshed with serum-reduced media (1% FBS). After 16 h, the cells were treated with increasing concentrations of the PIGA ligands (ranging from 10 nM to 1 µM) for 48 h. In the experiments used to evaluate the specific contribution of PIGA ligand-induced steroid production, the U87MG cells were pretreated (1 h before the addition of the PIGA ligands) with AMG (50 µM), a potent inhibitor of steroidogenesis. After 48 h, the MTS reagent was added to the PIGA ligand-treated cells, and the colorimetric MTS conversion was quantified after 2 h by measuring the absorbance at 490 nm with a microplate reader (WallacVictor 2, 1420 Multilabel Counter, Perkin Elmer, MA, USA).

### 4.5. Pregnenolone Quantification

The amount of pregnenolone in the rat C6 and human U87MG astrocyte models were quantified as previously reported [46]. Briefly, C6 or U87MG cells were incubated with 40 µM TSPO PIGA ligands in saline medium (140 mM NaCl, 5 mM KCl, 1.8 mM CaCl_2_, 1 mM MgSO_4_, 10 mM glucose, 10 mM *N*-2-hydroxyethylpiperazine-*N*′-2-ethanesulfonic acid (HEPES)–NaOH, pH 7.4, and 0.1% bovine serum albumin) containing the inhibitors of pregnenolone metabolism trilostane (25 μM) and SU10603 (10 μM). After 2 h of incubation, the conditioned salt medium was collected and the amount of pregnenolone secreted into the medium was quantified by an ELISA.

### 4.6. RT Determination of the TSPO PIGA Ligands

The RT values of the TSPO PIGA ligands were calculated from the reciprocal of their dissociation rate constant (*k*_off_). The *k*_off_ values were assessed by the competition kinetic association assay, as previously reported [46]. In particular, the TSPO-specific and TSPO-selective radioligand [^3^H]PK11195 (approximately 20 nM, specific activity of 21.4 μCi/nmol) and PIGA ligand were simultaneously added to the final reaction volume (500 µL) containing a rat kidney membrane homogenate (30 µg of proteins) and assay buffer (50 mM Tris-HCl, pH 7.4). The incubation of the samples was terminated at various times by vacuum filtration. The PIGA ligands were solubilized with DMSO and tested at a concentration corresponding to a three-fold higher value than the respective value of the inhibition constant (*K*_i_). The *K*_i_ values have been reported previously [52]. The nonspecific binding was determined in the presence of 1 µM PK11195. The experimentally derived data were analyzed using the “kinetics of competitive binding” using Prism 5.0 (GraphPad Software Inc., San Diego, CA, USA).

### 4.7. Radiolabel Binding Experiment in Human Astrocytes Using [^3^H] PK11195

For crude membranes, confluent human astrocytes derived from a 175 cm^2^ cell flask were harvested using phosphate-buffered saline (PBS), pH 7.4, supplemented with EDTA 0.04%. After the cells were collected by centrifugation (200× *g* for 5 min), the pellet was suspended in 10 mL of ice-cold buffer (5 mM Tris–HCl, pH 7.4 containing protease inhibitors (160 μg/mL benzamidine, 200 μg/mL bacitracin and 20 μg/mL trypsin inhibitor)) and homogenized with an Ultraturrax. Then, the homogenate was centrifuged at 48,000× *g* for 15 min at 4 °C and the supernatant was discarded. The obtained pellet was suspended in 10 mL of 50 mM Tris–HCl, pH 7.4 (assay buffer) containing the same amounts of protease inhibitors as described above, and the homogenate was pelleted by centrifugation (48,000× *g* for 15 min at 4 °C). The pellet was washed once with assay buffer and an additional centrifugation step was performed (48,000× *g* for 15 min at 4 °C). The resulting cell membrane pellet was suspended at a final concentration of 1 mg of protein/mL in assay buffer and used for the binding assays. The protein content of a 20 μL membrane suspension was measured by the Bradford method using the Bio-Rad Protein Assay reagent, according to the manufacturer’s protocol, with bovine serum albumin (BSA) as the standard.

To determine the specific binding of [^3^H]-PK11195 to the human astrocyte membrane suspensions, equilibrium radioligand binding assays were performed essentially as previously described [46,87]. Briefly, different aliquots of human astrocyte membranes (10–100 μg of proteins) were incubated with [^3^H]-PK11195 (1.5 nM) in the presence (non-specific binding) or absence (total binding) of unlabelled PK11195 (1 μM) in a final volume of 500 mL of assay buffer for 90 min at 0 °C. For the saturation experiments, aliquots of human astrocyte membranes (20 μg of proteins) were incubated with eight different concentrations of [^3^H]-PK11195 (0.5–30 nM) in duplicate using the conditions described above. In each assay, the final ethanol concentration in the incubation buffer was less than 1% and did not interfere with specific [^3^H]-PK11195 binding.

### 4.8. Statistical Analysis

The data are reported as the means ± SEM of at least three independent experiments. All statistical analyses were performed using GraphPad 5.0 Prism Software (GraphPad Software, La Jolla, CA, USA). One-way ANOVA with Bonferroni’s post-test and Spearman’s correlation analyses were used to assess the statistical significance of the data. A *p* value ≤ 0.05 was considered statistically significant.

## Figures and Tables

**Table 1:** TSPO binding affinity of the compounds. The concentration of the tested compounds that inhibited [^3^H]PK11195 binding to rat kidney mitochondrial membranes (IC_50_) by 50% was determined using six concentrations of the displacers, each performed in triplicate. The *K*_i_ values are the means ± SEM of three determinations.

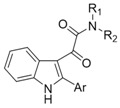	**Compound**	***R*_1_**	***R*_2_**	**Ar**	***K*_i_** **(nM) ^a^**
	**PIGA1228**	(CH_2_)_3_CH_3_	(CH_2_)_3_CH_3_	3-Thienyl	2.83 ± 0.30
	**PIGA1248**	(CH_2_)_5_CH_3_	(CH_2_)_5_CH_3_	3-Thienyl	0.89 ± 0.10
	**PIGA1175**	(CH_2_)_2_CH_3_	(CH_2_)_2_CH_3_	*p*-Biphenyl	0.53 ± 0.05
	**PIGA1165**	(CH_2_)_3_CH_3_	(CH_2_)_3_CH_3_	*p*-Biphenyl	5.50 ± 1.00
	**PIGA1174**	(CH_2_)_5_CH_3_	(CH_2_)_5_CH_3_	*p*-Biphenyl	1.84 ± 0.20
	**PIGA1128**	(CH_2_)_2_CH_3_	(CH_2_)_2_CH_3_	2-Naphthyl	0.30 ± 0.04
	**PIGA1136**	(CH_2_)_3_CH_3_	(CH_2_)_3_CH_3_	2-Naphthyl	0.53 ± 0.06
	**PIGA1130**	(CH_2_)_5_CH_3_	(CH_2_)_5_CH_3_	2-Naphthyl	0.52 ± 0.06
	**PIGA1137**	CH_3_	(CH_2_)_3_CH_3_	2-Naphthyl	0.56 ± 0.06
	**PIGA1138**	CH_3_	(CH_2_)_4_CH_3_	2-Naphthyl	0.37 ± 0.04
	**PIGA1226**	(CH_2_)_3_CH_3_	(CH_2_)_3_CH_3_	*p*-Methoxyphenyl	20.3 ± 2.21
	**PIGA1244**	(CH_2_)_5_CH_3_	(CH_2_)_5_CH_3_	*p*-Methoxyphenyl	4.04 ± 0.44
	**PIGA1212** ^a^	(CH_2_)_2_CH_3_	(CH_2_)_2_CH_3_	*p*-Methylphenyl	5.50 ± 0.38
	**PK 11195 ^b^**				9.3 ± 0.50

^a^ Data taken from ref. [52]; ^b^ Data taken from ref. [51].

**Table 2:** Pregnenolone production in C6 and U87MG cells. The data are expressed as percentage of pregnenolone production compared to the control, which was set to 100%, and represent the means ± SEM of three different determinations performed in duplicate. The statistical analysis was performed using one-way ANOVA and Bonferroni’s post-test.

Compounds	Pregnenolone Production in C6 cells (% ± SEM)	*p*	Pregnenolone Production in U87MG cells (% ± SEM)	*p*
**PIGA1128**	148.4 ± 4.643	<0.01	190.0 ± 8.356	<0.001
**PIGA1136**	164.0 ± 2.096	<0.01	177.3 ± 7.963	<0.001
**PIGA1130**	155.0 ± 5.774	<0.01	145.8 ± 3.393	<0.01
**PIGA1137**	208.0 ± 4.933	<0.001	287.6 ± 11.98	<0.001
**PIGA1138**	245.0 ± 5.774	<0.001	298.7 ± 10.14	<0.001
**PK 11195**	138.5 ± 9.717	<0.05	143.8 ± 20.40	<0.01

## References

[1] Rupprecht R., Holsboer F. (1999). Neuroactive steroids: Mechanisms of action and neuropsychopharmacological perspectives. Trends Neurosci..

[2] Reddy D.S. (2010). Neurosteroids: Endogenous role in the human brain and therapeutic potentials. Prog. Brain Res..

[3] Pfaff D.W., Gerlach J.L., McEwen B.S., Ferin M., Carmel P., Zimmerman E.A. (1976). Autoradiographic localization of hormone-concentrating cells in the brain of the female rhesus monkey. J. Comp. Neurol..

[4] Buisson B., Bertrand D., Baulieu E.E., Robel P., Schumacher M. (1999). Steroid modulation of the nicotinic acetylcholine receptor. Neurosteroids: A New Regulatory Function in the Nervous System.

[5] Gibbs T.T., Yaghoubi N., Weaver C.E., Park-Chung M., Russek S.J., Farb D.H., Baulieu E.E., Robel P., Schumacher M. (1999). Modulation of ionotropic glutamate receptors by neuroactive steroids. Neurosteroids: A New Regulatory Function in the Nervous System.

[6] Majewska M.D., Baulieu E.E., Robel P., Schumacher M. (1999). Neurosteroid antagonists of the GABAA receptors. Neurosteroids: A New Regulatory Function in the Nervous System.

[7] Bastianetto S., Ramassamy C., Poirier J., Quirion R. (1999). Dehydroepiandrosterone (DHEA) protects hippocampal cells from oxidative stress-induced damage. Mol. Brain Res..

[8] Gee K.W., McCaule L.D., Lan N.C. (1995). A putative receptor for neurosteroids on the GABAA receptor complex: The pharmacological properties and therapeutic potential of epalons. Crit. Rev. Neurobiol..

[9] Ueda H., Yoshida A., Tokuyama S., Mizuno K., Maruo J., Matsuno K., Mita S. (2001). Neurosteroids stimulate G protein-coupled sigma receptors in mouse brain synaptic membrane. Neurosci. Res..

[10] Schlichter R., Keller A.F., de Roo M., Breton J.D., Inquimbert P., Poisbeau P. (2006). Fast nongenomic effects of steroids on synaptic transmission and role of endogenous neurosteroids in spinal pain pathways. J. Mol. Neurosci..

[11] Herd M.B., Belelli D., Lambert J.J. (2007). Neurosteroid modulation of synaptic and extrasynaptic GABA(A) receptors. Pharmacol. Ther..

[12] Magnaghi V. (2007). GABA and neuroactive steroid interactions in glia: New roles for old players?. Curr. Neuropharmacol..

[13] Duenas M., Luquin S., Chowen J.A., Torres-Aleman I., Naftolin F., Garcia-Segura L.M. (1994). Gonadal hormone regulation of insulin-like growth factor-I-like immunoreactivity in hypothalamic astroglia of developing and adult rats. Neuroendocrinology.

[14] Kirschner P.B., Henshaw R., Weise J., Trubetskoy V., Finklestein S., Schulz J.B., Beal M.F. (1995). Basic fibroblast growth factor protects against excitotoxicity and chemical hypoxia in both neonatal and adult rats. J. Cereb. Blood Flow Metab..

[15] Stone D.J., Rozovsky I., Morgan T.E., Anderson C.P., Hajian H., Finch C.E. (1997). Astrocytes and microglia respond to estrogen with increased apoE mRNA in vivo and in vitro. Exp. Neurol..

[16] Flores C., Salmaso N., Cain S., Rodaros D., Stewart J. (1999). Ovariectomy of adult rats leads to increased expression of astrocytic basic fibroblast growth factor in the ventral tegmental area and in dopaminergic projection regions of the entorhinal and prefrontal cortex. J. Neurosci..

[17] Buchanan C.D., Mahesh V.B., Brann D.W. (2000). Estrogen-astrocyte-luteinizing hormone-releasing hormone signaling: A role for transforming growth factor-β1. Biol. Reprod..

[18] Galbiati M., Martini L., Melcangi R.C. (2002). Oestrogens, via transforming growth factor α, modulate basic fibroblast growth factor synthesis in hypothalamic astrocytes: In vitro observations. J. Neuroendocrinol..

[19] Kazanis I., Giannakopoulou M., Philippidis H., Stylianopoulou F. (2004). Alterations in IGF-I, BDNF and NT-3 levels following experimental brain trauma and the effect of IGF-I administration. Exp. Neurol..

[20] Platania P., Seminara G., Aronica E., Troost D., Vincenza Catania M., Angela Sortino M. (2005). 17β-estradiol rescues spinal motoneurons from AMPA-induced toxicity: A role for glial cells. Neurobiol. Dis..

[21] Mendez P., Cardona-Gomez G.P., Garcia-Segura L.M. (2005). Interactions of insulin-like growth factor-I and estrogen in the brain. Adv. Exp. Med. Biol..

[22] Dhandapani K.M., Brann D.W. (2007). Role of astrocytes in estrogen-mediated neuroprotection. Exp. Gerontol..

[23] Cerciat M., Unkila M., Garcia-Segura L.M., Arevalo M.A. (2010). Selective estrogen receptor modulators decrease the production of interleukin-6 and interferon-gamma-inducible protein-10 by astrocytes exposed to inflammatory challenge in vitro. Glia.

[24] Xu S.L., Bi C.W., Choi R.C., Zhu K.Y., Miernisha A., Dong T.T., Tsim K.W. (2013). Flavonoids induce the synthesis and secretion of neurotrophic factors in cultured rat astrocytes: A signaling response mediated by estrogen receptor. Evid. Based Complement. Altern. Med..

[25] Spence R.D., Wisdom A.J., Cao Y., Hill H.M., Mongerson C.R., Stapornkul B., Itoh N., Sofroniew M.V., Voskuhl R.R. (2013). Estrogen mediates neuroprotection and anti-inflammatory effects during EAE through ERα signaling on astrocytes but not through ERβ signaling on astrocytes or neurons. J. Neurosci..

[26] Lee E., Sidoryk-Wêgrzynowicz M., Wang N., Webb A., Son D.S., Lee K., Aschner M. (2012). GPR30 regulates glutamate transporter GLT-1 expression in rat primary astrocytes. J. Biol. Chem..

[27] Araújo G.W., Beyer C., Arnold S. (2008). Oestrogen influences on mitochondrial gene expression and respiratory chain activity in cortical and mesencephalic astrocytes. J. Neuroendocrinol..

[28] Irwin R.W., Yao J., Hamilton R.T., Cadenas E., Brinton R.D., Nilsen J. (2008). Progesterone and estrogen regulate oxidative metabolism in brain mitochondria. Endocrinology.

[29] Morohaku K., Pelton S.H., Daugherty D.J., Butler W.R., Deng W., Selvaraj V. (2014). Translocator protein/peripheral benzodiazepine receptor is not required for steroid hormone biosynthesis. Endocrinology.

[30] Tu L.N., Morohaku K., Manna P.R., Pelton S.H., Butler W.R., Stocco D.M., Selvaraj V. (2014). Peripheral benzodiazepine receptor/translocator protein global knock-out mice are viable with no effects on steroid hormone biosynthesis. J. Biol. Chem..

[31] Banati R.B., Middleton R.J., Chan R., Hatty C.R., Kam W.W., Quin C., Graeber M.B., Parmar A., Zahra D., Callaghan P. (2014). Positron emission tomography and functional characterization of a complete PBR/TSPO knockout. Nat. Commun..

[32] Papadopoulos V., Lecanu L. (2009). Translocator protein (18 kDa) TSPO: An emerging therapeutic target in neurotrauma. Exp. Neurol..

[33] Frye C.A. (2009). Neurosteroids’ effects and mechanisms for social, cognitive, emotional, and physical functions. Psychoneuroendocrinology.

[34] Fan J., Campioli E., Midzak A., Culty M., Papadopoulos V. (2015). Conditional steroidogenic cell-targeted deletion of TSPO Unveils a Crucial role in Viability and Hormone-Dependent Steroid Formation. Proc. Natl. Acad. Sci. USA.

[35] Mellon S.H., Deschepper C.F. (1993). Neurosteroid biosynthesis: Genes for adrenal steroidogenic enzymes are expressed in the brain. Brain Res..

[36] King S.R., Ginsberg S.D., Ishii T., Smith R.G., Parker K.L., Lamb D.J. (2004). The steroidogenic acute regulatory protein is expressed in steroidogenic cells of the day-old brain. Endocrinology.

[37] Papadopoulos V., Baraldi M., Guilarte T.R., Knudsen T.B., Lacapère J.J., Lindemann P., Norenberg M.D., Nutt D., Weizman A., Zhang M.R. (2006). Translocator protein (18 kDa): New nomenclature for the peripheral-type benzodiazepine receptor based on its structure and molecular function. Trends Pharmacol. Sci..

[38] Lacapère J.J., Papadopoulos V. (2003). Peripheral-type benzodiazepine receptor: Structure and function of a cholesterol-binding protein in steroid and bile acid biosynthesis. Steroids.

[39] Da Pozzo E., Costa B., Martini C. (2012). Translocator protein (TSPO) and neurosteroids: Implications in psychiatric disorders. Curr. Mol. Med..

[40] Da Pozzo E., Giacomelli C., Barresi E., Costa B., Taliani S., da Settimo F., Martini C. (2015). Targeting the 18 kDa translocator protein: Recent perspectives for neuroprotection. Biochem. Soc. Trans..

[41] Veiga S., Azcoitia I., Garcia-Segura L.M. (2005). Ro5-4864, a peripheral benzodiazepine receptor ligand, reduces reactive gliosis and protects hippocampal hilar neurons from kainic acid excitotoxicity. J. Neurosci. Res..

[42] Veiga S., Carrero P., Pernia O., Azcoitia I., Garcia-Segura L.M. (2007). Translocator protein 18 kDa is involved in the regulation of reactive gliosis. Glia.

[43] Girard C., Liu S., Cadepond F., Adams D., Lacroix C., Verleye M., Gillardin J.M., Baulieu E.E., Schumacher M., Schweizer-Groyer G. (2008). Etifoxine improves peripheral nerve regeneration and functional recovery. Proc. Natl. Acad. Sci. USA.

[44] Zhao Y.Y., Yu J.Z., Li Q.Y., Ma C.G., Lu C.Z., Xiao B.G. (2011). TSPO-specific ligand vinpocetine exerts a neuroprotective effect by suppressing microglial inflammation. Neuron Glia Biol..

[45] Santoro A., Mattace Raso G., Taliani S., da Pozzo E., Simorini F., Costa B., Martini C., Laneri S., Sacchi A., Cosimelli B. (2016). TSPO-ligands prevent oxidative damage and inflammatory response in C6 glioma cells by neurosteroid synthesis. Eur. J. Pharm. Sci..

[46] Costa B., da Pozzo E., Giacomelli C., Barresi E., Taliani S., da Settimo F., Martini C. (2016). TSPO ligand residence time: A new parameter to predict compound neurosteroidogenic efficacy. Sci. Rep..

[47] Chen J.H., Tsou T.C., Chiu I.M., Chou C.C. (2010). Proliferation inhibition, DNA damage, and cell-cycle arrest of human astrocytoma cells after acrylamide exposure. Chem. Res. Toxicol..

[48] Li Y., Cheng D., Cheng R., Zhu X., Wan T., Liu J., Zhang R. (2014). Mechanisms of U87 astrocytoma cell uptake and trafficking of monomeric versus protofibril Alzheimer’s disease amyloid-β proteins. PLoS ONE.

[49] Maresca B., Spagnuolo M.S., Cigliano L. (2015). Haptoglobin modulates β-amyloid uptake by U-87 MG astrocyte cell line. J. Mol. Neurosci..

[50] Munoz L., Kavanagh M.E., Phoa A.F., Heng B., Dzamko N., Chen E.J., Doddareddy M.R., Guillemin G.J., Kassiou M. (2015). Optimisation of LRRK2 inhibitors and assessment of functional efficacy in cell-based models of neuroinflammation. Eur. J. Med. Chem..

[51] Da Settimo F., Simorini F., Taliani S., La Motta C., Marini A.M., Salerno S., Bellandi M., Novellino E., Greco G., Cosimelli B. (2008). Anxiolytic-like effects of *N*,*N*-dialkyl-2-phenylindol-3-ylglyoxylamides by modulation of translocator protein promoting neurosteroid biosynthesis. J. Med. Chem..

[52] Barresi E., Bruno A., Taliani S., Cosconati S., da Pozzo E., Salerno S., Simorini F., Daniele S., Giacomelli C., Marini A.M. (2015). Deepening the topology of the Translocator Protein binding site by novel *N*,*N*-dialkyl-2-arylindol-3-ylglyoxylamides. J. Med. Chem..

[53] Kugler W., Veenman L., Shandalov Y., Leschiner S., Spanier I., Lakomek M., Gavish M. (2008). Ligands of the mitochondrial 18 kDa translocator protein attenuate apoptosis of human glioblastoma cells exposed to erucylphosphohomocholine. Cell Oncol..

[54] Banfalvi G. (2011). Cell Cycle Synchronization. Methods and Protocols.

[55] Chen M., Huang J., Yang X., Liu B., Zhang W., Huang L., Deng F., Ma J., Bai Y., Lu R., Huang B., Gao Q., Zhuo Y., Ge J. (2012). Serum starvation induced cell cycle synchronization facilitates human somatic cells reprogramming. PLoS ONE.

[56] Takahashi S., Abe T., Gotoh J., Fukuuchi Y. (2002). Substrate-dependence of reduction of MTT: A tetrazolium dye differs in cultured astroglia and neurons. Neurochem. Int..

[57] Dunigan D.D., Waters S.B., Owen T.C. (1995). Aqueous soluble tetrazolium/formazan MTS as an indicator of NADH- and NADPH-dependent dehydrogenase activity. Biotechniques.

[58] Mosmann T. (1983). Rapid colorimetric assay for cellular growth and survival: Application to proliferation and cytotoxicity assays. J. Immunol. Methods.

[59] Papadopoulos V., Guarneri P., Kreuger K.E., Guidotti A., Costa E. (1992). Pregnenolone biosynthesis in C6-2B glioma cell mitochondria: Regulation by a mitochondrial diazepam binding inhibitor receptor. Proc. Natl. Acad. Sci. USA.

[60] Azcoitia I., Sierra A., Veiga S., Garcia-Segura L.M. (2003). Aromatase expression by reactive astroglia is neuroprotective. Ann. N. Y. Acad. Sci..

[61] Groeneveld G.J., van Muiswinkel F.L., Sturkenboom J.M., Wokke J.H., Bär P.R., van den Berg L.H. (2004). Ovariectomy and 17β-estradiol modulate disease progression of a mouse model of ALS. Brain Res..

[62] Garcia-Ovejero D., Azcoitia I., Doncarlos L.L., Melcangi R.C., Garcia-Segura L.M. (2005). Glia-neuron crosstalk in the neuroprotective mechanisms of sex steroid hormones. Brain Res. Rev..

[63] Conejo N.M., González-Pardo H., Cimadevilla J.M., Argüelles J.A., Díaz F., Vallejo-Seco G., Arias J.L. (2005). Influence of gonadal steroids on the glial fibrillary acidic protein-immunoreactive astrocyte population in young rat hippocampus. J. Neurosci. Res..

[64] Barreto G., Veiga S., Azcoitia I., Garcia-Segura L.M., Garcia-Ovejero D. (2007). Testosterone decreases reactive astroglia and reactive microglia after brain injury in male rats: Role of its metabolites, oestradiol and dihydrotestosterone. Eur. J. Neurosci..

[65] Choi C.I., Lee Y.D., Gwag B.J., Cho S.I., Kim S.S., Suh-Kim H. (2008). Effects of estrogen on lifespan and motor functions in female hSOD1 G93A transgenic mice. J. Neurol. Sci..

[66] Garcia-Segura L.M. (2009). Hormones and Brain Plasticity.

[67] Arevalo M.A., Santos-Galindo M., Bellini M.J., Azcoitia I., Garcia-Segura L.M. (2010). Actions of estrogens on glial cells: Implications for neuroprotection. Biochim. Biophys. Acta.

[68] Gandolfo P., Patte C., Thoumas J.L., Leprince J., Vaudry H., Tonon M.C. (1999). The endozepine ODN stimulates [3H]thymidine incorporation in cultured rat astrocytes. Neuropharmacology.

[69] Gandolfo P., Patte C., Leprince J., Régo J.L., Mensah-Nyagan A.G., Vaudry H., Tonon M.C. (2000). The triakontatetraneuropeptide (TTN) stimulates thymidine incorporation in rat astrocytes through peripheral-type benzodiazepine receptors. J. Neurochem..

[70] Neary J.T., Jorgensen S.L., Oracion A.M., Bruce J.H., Norenberg M.D. (1995). Inhibition of growth factor-induced DNA synthesis in astrocytes by ligands of peripheral-type benzodiazepine receptors. Brain Res..

[71] Bruce J.H., Ramirez A.M., Lin L., Oracion A., Agarwal R.P., Norenberg M.D. (1991). Peripheral-type benzodiazepines inhibit proliferation of astrocytes in culture. Brain Res..

[72] Ikezaki K., Black K.L. (1990). Stimulation of cell growth and DNA synthesis by peripheral benzodiazepine. Cancer Lett..

[73] Miccoli L., Oudard S., Beurdeley-Thomas A., Dutrillaux B., Poupon M.F. (1999). Effect of 1-(2-chlorophenyl)-*N*-methyl-*N*-(1-methylpropyl)-3-isoquinoline carboxamide (PK11195), a specific ligand of the peripheral benzodiazepine receptor, on the lipid fluidity of mitochondria in human glioma cells. Biochem. Pharmacol..

[74] Shiraishi T., Black K.L., Ikezaki K., Becker D.P. (1991). Peripheral benzodiazepine induces morphological changes and proliferation of mitochondria in glioma cells. J. Neurosci. Res..

[75] Black K.L., Shiraishi T., Ikezak K., Tabuchi K., Becker D.P. (1994). Peripheral benzodiazepine stimulates secretion of growth hormone and mitochondrial proliferation in pituitary tumour GH3 cells. Neurol. Res..

[76] Scarf A.M., Auman K.M., Kassiou M. (2012). Is there any correlation between binding and functional effects at the translocator protein (TSPO) (18 kDa)?. Curr. Mol. Med..

[77] Alho H., Varga V., Krueger K.E. (1994). Expression of mitochondrial benzodiazepine receptor and its putative endogenous ligand diazepam binding inhibitor in cultured primary astrocytes and C-6 cells: Relation to cell growth. Cell Growth Differ..

[78] Gao Z.W., Huang J.B., Lin Q., Qin Q., Liang Y.J., Zhou L., Luo M. (2016). The effects of PK11195 on meningioma was associated with allopregnanolone biosynthesis, which was mediated by translocator protein 18 kDa. Cancer Biomark..

[79] Hechter O., Halkerston I.D. (1965). Effects of steroid hormones on gene regulation and cell metabolism. Annu. Rev. Physiol..

[80] Takuma K., Baba A., Matsuda T. (2004). Astrocyte apoptosis: Implications for neuroprotection. Prog. Neurobiol..

[81] Wagner B., Natarajan A., Grünaug S., Kroismayr R., Wagner E.F., Sibilia M. (2006). Neuronal survival depends on EGFR signaling in cortical but not midbrain astrocytes. EMBO J..

[82] Bauer J., Elger C.E., Hans V.H., Schramm J., Urbach H., Lassmann H., Bien C.G. (2007). Astrocytes are a specific immunological target in Rasmussen’s encephalitis. Ann. Neurol..

[83] Ricci G., Volpi L., Pasquali L., Petrozzi L., Siciliano G. (2009). Astrocyte-neuron interactions in neurological disorders. J. Biol. Phys..

[84] Wingerchuk D.M. (2010). Neuromyelitis optica spectrum disorders. Continuum (Minneap. Minn.).

[85] Escartin C., Rouach N. (2013). Astroglial networking contributes to neurometabolic coupling. Front. Neuroenerg..

[86] Najjar S., Pearlman D.M., Alper K., Najjar A., Devinsky O. (2013). Neuroinflammation and psychiatric illness. J. Neuroinflamm..

[87] Phatnani H., Maniatis T. (2015). Astrocytes in neurodegenerative disease. Cold Spring Harb. Perspect. Biol..

